# Testing Links of Food-Related Olfactory Perception to Peripheral Ghrelin and Leptin Concentrations

**DOI:** 10.3389/fnut.2022.888608

**Published:** 2022-05-11

**Authors:** Rachel Ginieis, Sashie Abeywickrema, Indrawati Oey, Mei Peng

**Affiliations:** ^1^Sensory Neuroscience Laboratory, Department of Food Science, University of Otago, Dunedin, New Zealand; ^2^Riddet Institute, Palmerston North, New Zealand

**Keywords:** ghrelin, leptin, obesity, olfaction, food odour

## Abstract

The peptide hormones ghrelin and leptin play major roles in the regulation of appetite and food intake. However, the precise effects of these hormones on sensory processing remain a subject of debate, particularly with food related stimuli and its small body of evidence. Here, we test for relationships between ghrelin and leptin levels against olfactory performance with multiple food-related odours. Specifically, a total of 94 Caucasian males were tested for their supra-threshold sensitivity (i.e., d′), intensity, and valence perception to three odour compounds (i.e., *vanilla*, *potato*, and *dairy* odours). These sensory data were then analysed against peripheral ghrelin and leptin levels, both assessed in plasma samples. Participants’ body adiposity measures were also obtained. Results lent strong support to one of our original hypotheses, with ghrelin levels being positively correlated to the supra-threshold sensitivity of the *dairy* odour, (*r* = 0.241, *p* = 0.020), and intensity ratings to most of the food odours tested [*dairy* (*r* = 0.216, *p* = *0.037*) and *vanilla* (*r* = 0.241, *p* = *0.020*)]. By contrast, peripheral leptin levels were not significantly linked to any of the olfactory measures (*p* > 0.05). These relationships remained similar after controlling for variabilities of adiposity measures. The present study brings novel insights by identifying positive links between supra-threshold olfactory perception and ghrelin. This new knowledge is highly relevant for future research linking olfactory shifts to hormonal dysregulation and obesity.

## Introduction

Understanding the aetiology of obesity remains an important research direction (1). Over-responsiveness to food cues is considered a key contributor to obesity in the current food environment (2, 3). Research over the last two decades has shown that maladaptive eating behaviour is often accompanied by major alterations in peptide hormones, such as ghrelin and leptin (4, 5). However, mechanisms underpinning these observed relationships remain unclear. Recent research has postulated that sensory processing plays an important role in mediating hormonal effects on eating (6–8). The current study adds to the emerging body of research by testing for links between peripheral leptin and ghrelin levels and olfactory perception of food-related stimuli.

Previous research has consistently observed links between obesity and resistance to leptin and ghrelin [e.g., (9, 10)]. Notably, peripheral ghrelin and leptin have been increasingly used as biomarkers for obesity (9, 11). Individuals with obesity were shown to have reduced ghrelin (1/3–1/2 time lower) (11, 12), and increased leptin levels (2–8 times higher), compared to normal-weight controls (13–15). In addition to links to obesity, neurological evidence suggests that both ghrelin and leptin can modulate neural responsiveness to food rewards (4, 5, 16). Specifically, ghrelin is an orexigenic agent (i.e., promoting food intake) (17, 18) and leptin is an anorexigenic agent (i.e., inhibiting food intake) (19, 20). Based on these previous findings, it is intuitive to propose that changes of ghrelin and leptin alter food-related neurological and behavioural responses, and correspondingly influence one’s body weight overtime (5, 21–23). However, the precise mechanism underpinning these effects remains unclear. Recent research suggests that sensory processing may play a key role in mediating ghrelin and leptin effects on eating [cf. (24)], although such findings remain controversial.

Across the five special senses, olfaction is the least understood, despite its vital function in flavour perception and food acceptance (25). Olfactory processing emerges from first-order neurons at the olfactory mucosa (OM) toward the olfactory bulb (OB) (25). The OB then conveys olfactory information to the olfactory cortex (26–28), which includes the piriform cortex, anterior olfactory nucleus, lateral entorhinal cortex, periamygdaloid cortex and the cortical nucleus of the amygdala (26, 29–31). Further higher order projections from the olfactory cortex to the orbitofrontal cortex, amygdala, and hippocampus encode for executive, emotional, motivational, and memory-related processes associated with human olfaction (32). Thus, higher-order processing confers its specificity to the stimulus perceived and reveal odour features, including odour intensity and valence (29). This temporal cascade of olfactory processes is shown to start with odour detection and discrimination, followed by the identification of odour quality (e.g., the smell of a rose) and ends with the hedonic perception of this stimulus (33). Altogether, these complex neuroanatomical pathways govern different aspects of an individual’s olfactory perception, from detection sensitivity to hedonic valence, highlighting a fundamental difference between these sensory measures.

Ghrelin and leptin can traverse the blood-brain barrier and reach several cerebral areas that are directly involved with feeding behaviour [e.g., hypothalamus: (34–36); mesolimbic reward system: (37)], as well as other sensory-related regions that are indirectly linked to eating (38–40). Recent research further indicated that ghrelin and leptin were particularly involved in olfactory transduction (41). Specifically, ghrelin receptors are found in olfactory structures such as the glomeruli, mitral cells, and granule cells located in the OB (39). More recent research indicated that ghrelin is able to modulate olfactory information transmission from the mitral cells to the amygdala and hypothalamus (42). Similarly, leptin and its associated receptor have been found in the OM (43), olfactory epithelium (44) and OB (45). More functional evidence indicated that leptin was involved in olfactory-related mechanisms such as the mucus production (41, 46). Furthermore, ghrelin and leptin signalling, and olfactory transduction were shown to be co-modulated in shared cerebral structures that are closely related to feeding (35, 42). These observed links point to the possibility that ghrelin and leptin influence eating behaviour *via* shaping individual olfactory perception.

A few studies have tested for links between olfactory perception and peripheral ghrelin levels, with findings remaining controversial. Specifically, a subset of these studies attempted to test for links between peripheral ghrelin and detection threshold to neutral odours (e.g., n-butanol) (47–50). However, most of these studies failed to detect any significant relationship (47–49). Only a recent study found a significant positive correlation between peripheral ghrelin levels and detection threshold scores to *n-butanol* (50). Importantly, the study from Uygun et al. (50) was performed only on women with obesity while other studies considered all weight groups for analysis, which may have contributed to these differential results. Although no study tested for links between peripheral ghrelin and olfactory sensitivity to food odours, a few studies showed that systemic ghrelin infusions significantly increased individual sniffing magnitude in response to food odours (39) and generated a greater response to food odour conditioning task (48). In line with these findings, peripheral ghrelin levels were shown to be significantly associated with valence ratings (VR) of food odours (e.g., black pepper oil) (48, 49). Such inter-study inconsistencies may indicate the important role of the nature of odorants in the relationship between peripheral ghrelin and olfaction.

With regards to the link between leptin and olfactory acuity, animal studies have consistently observed a negative relationship [e.g., (51)], while human studies revealed mixed findings. Specifically, several rodent studies observed that leptin-deficient animals exhibited a heightened sensitivity to food-related odours, compared to matched controls (44, 52, 53). Such findings were later explained as leptin reduces neural activity in the olfactory epithelium and OB (45, 54–56). Accordingly, a recent human study highlighted a negative correlation between OB volume and peripheral leptin levels (57). On the other hand, studies with human subjects regarding olfactory sensitivity revealed either positive (49, 58) or negative correlations with peripheral leptin levels (58, 59). Karlsson et al. (58) further pointed out differential results being dependent on the sex of participants with peripheral leptin levels and olfactory sensitivity being negatively linked in females and positively linked in males. Notably, these studies mainly focussed on identification, detection and discrimination of a neutral olfactory compound, *n-butanol*. The study from Trellakis et al. (49) was the only experiment on human subjects incorporating food odours, and their results revealed a significant correlation between pleasantness to black pepper oil smell and peripheral levels of leptin. Given that numerous animal studies using food odours observed a consistent negative relationship between leptin and olfaction, the use of food odours to assess olfactory functions in humans may reveal a consistent link with peripheral leptin levels.

The present paper aims to test for links of peripheral ghrelin and leptin levels to olfactory functions. Due to the close relationship between olfactory supra-threshold functions and eating behaviour [e.g., (60)], the present study compares peripheral hormone levels to supra-threshold sensitivities, intensity ratings (IR), and VR to three food-related odours. Building upon previous literature, we hypothesised that individuals with elevated levels of peripheral ghrelin show heightened olfactory supra-threshold sensitivity and IR, but reduced VR of food odours. By contrast, we hypothesised that individuals with high levels of peripheral leptin levels show weakened olfactory supra-threshold sensitivity and IR, but heightened VR for food odours.

## Materials and Methods

### Participants

A total of 94 Caucasian males [25.2 ± 5.7 years of age; body mass index (BMI): 26.7 ± 4.9 kg⋅m^–2^] undertook this study. Given the high degree of inter-individual differences in olfactory perception due to sex, only males were recruited and included in the present study [cf. (61)]. Sample size was determined using G*Power 3.1.9.7 Software, with calculations being based on the effect sizes reported in previous studies testing for links between peripheral hormone levels and olfactory performances [e.g., (49, 50)]. The analysis produced a sample size of 84 to achieve an 80% power and an α-level of 0.05, based on bivariate normal model of correlations (effect size *r* = 0.300). We decided to recruit an additional 10 participants to allow for at least 10% of predicted rate of participants’ withdrawal. All participants were non-smokers, and were free from sensory dysfunctions, chronic medical conditions, or food allergies. Participants were required to abstain from food or non-water beverage after 10 p.m. of the night prior to each laboratory session and the phlebotomy appointment. In addition, participants were asked not to wear any cologne or scented cosmetic on the day of the testing. All participants gave informed written consents. The study was approved by the University of Otago Ethics Committee for Human Participation – health panel (Reference: H18/111). Each participant received a monetary compensation upon completion of the study.

### Study Overview

Each participant attended six 30-min experimental sessions over consecutive weekdays, from 7.00 to 9.30 a.m., and a separate phlebotomy test for blood sample collection. All experimental sessions were carried out in standard individual sensory booths, at 20°C and under red light, in the Sensory Neuroscience Laboratory at the University of Otago, New Zealand. The six experimental sessions included replicated assessments of individual supra-threshold sensitivities, intensity, and valence perception of three food-related odours. Testing orders were randomised across participants using a Latin Square design (62). Participants’ weight and height were measured in laboratory for calculating anthropometric measures. On a separate morning after the completion of sensory tests, participants were asked to attend a phlebotomy appointment for collection of a fasting blood sample.

### Stimuli

Information of the olfactory stimuli used in this study are described in Table 1. These odorants were selected due to their close relevance to common snack foods in New Zealand (63, 64). Each odorant was made into solutions of 11 concentrations following an additive logarithmic steps, with the middle concentration being the reference sample. The five lower concentration levels referred to the *decremental series*, and the five higher concentration levels to the *incremental series*. Serial dilution method was used to make these solutions, with filtered water (0.5 μm) being the solvent. According to previous reports and pilot tests, the selected concentration range of the olfactory compounds should be above the recognition thresholds associated with the odorant (60, 65, 66). During the sensory tests, all olfactory samples were presented at a volume of 5 mL in 50 mL glass bottles (73 mm height, 42 mm diameter, Arthur Holmes, New Zealand).

**TABLE 1:** Summary of olfactory stimuli characteristics including description, compound, suppliers, purity, and reference concentrations.

Odorant code	Odour descriptor	Chemical name	CAS number	Purity	Supplier	Reference (Concentration/log value)	Dilution log step
O1	Vanilla	4-Hydroxy-3-methoxybenzaldehyde *Vanillin*	121–33–5	> 99%	Vanesse, Camlin Fine Sciences, India	0.39 g⋅L^–1^ /–0.40	0.221
O2	Potato	3-(Methylthio) Propionaldehyde *Methional*	3268–49–3	> 96%	Sigma–Aldrich United States	1.86 × 10^–4^ mL⋅L^–1^ /–3.73	0.146
O3	Dairy	4h-Pyran-4-One, 3-Hydroxy-2-Methyl-	118–71–8	–	Firmenich, Switzerland	0.08 mL⋅L^–1^ /–1.10	0.221
		3(2h)-Furanone, 4-Hydroxy-2,5-Dimethyl- *Maltol/Furaneol* mixture	3658–77–3				

### Olfactory Supra-Threshold Sensitivity Measures

Each odorant was tested twice over two 30-min sessions on separate days. The supra-threshold sensitivity test was constructed based on the method of constant stimuli, with a two-alternative forced choice (2-AFC) presentation. In a single 2-AFC task, the participant was presented with one reference sample and one testing sample (from either the *incremental* or *decremental series*), following a pre-determined randomised order. The participants were required to sniff the two samples for two seconds each and then indicate the “most intense sample.” Each testing session contains 50 2-AFC comparisons, comprising five replicated testing of each concentration level. Across the two sessions, for each odorant, each concentration level was compared to the reference sample for 10 replicates. Participants were given non-flavoured crackers and a glass of water for inter-trial palate cleansing. Olfactory supra-threshold sensitivity tests were performed on *Compusense* Cloud software (Guelph, ON, Canada).

### Olfactory Intensity and Valence Ratings

Intensity and hedonic general Labelled Magnitude Scale (gLMS) were used to measure intensity and valence perception to each testing odorant. The intensity gLMS is a 100-point scale, marked with semi-logarithmically spaced descriptors (*no sensation* = 0, *weak* = 6, *moderate* = 17, *strong* = 35, *very strong* = 53, *strongest imaginable sensation of any kind* = 100) (67). The hedonic gLMS is a double scale ranging from −100 to 100, where −100 represents the *strongest imaginable dislike*, and 100 represents the *strongest imaginable like* (68). Other interval labels are similar to the intensity gLMS. Both scales are widely accepted and were shown to be adapted to scale olfactory sensations (67–69). On the first day of the six laboratory sessions, participants were instructed on how to use these scales (69). For each odorant, intensity and valence were rated against the reference sample (see Table 1). These ratings were performed on *Compusense* Cloud software (Guelph, ON, Canada).

### Measurements of Hormones

Blood samples were collected between 7.30 and 10 a.m. following overnight fasting. Samples were then centrifuged in a refrigerated centrifuge (4°C) for 10 min at 1,000 × g within 30 min of blood collection. The plasma was pipetted and transferred in K_2_ EDTA tubes and stored at −80°C until analysis. Leptin and acyl ghrelin (active ghrelin) were both measured in duplicate using a commercially available multiplex kit (Milliplex Map kit, Human Metabolic Hormone Magnetic Bead Panel HMHEMAG-34K; Millipore Corp., St. Louis, MO, United States). Milliplex map kits are specific to the Luminex Magpix Analyzer and are analytically validated for sensitivity, specificity, and reproducibility. These kits offer a great sensitivity for active ghrelin and leptin with respective detection thresholds of 14 pg/mL (intra-assay CV < 10%, inter-assay CV < 15%) and 41 pg/mL (intra-assay CV < 10%, inter-assay CV < 15%). Additionally, when a sample contained a lower concentration than the lowest detection threshold, standard curves were extrapolated to determine the sample concentration. The measurements of the 94 blood samples required three Milliplex map kits in total. The protocol was performed over two consecutive days, and the exact same procedure was followed for each kit.

### Anthropometric Measurements

Participants’ weight and height were measured using a standard scale and stadiometer to the nearest 0.1 unit. Participants were asked to stay in standing position wearing light clothing without shoes. Participants’ BMI were classified as normal weight for a value between 18.5 and 24.9 kg⋅m^–2^, overweight from 25 to 29.9 kg⋅m^–2^, and obese over 30 kg⋅m^–2^. Additionally, body fat percentage was also measured using skinfold thickness measurements on four body sites (biceps, triceps, subscapular area, and suprailiac area) as a complementary measure of BMI.

### Sensitivity Calculations

Individual supra-threshold sensitivities were calculated based on the results obtained from 2-AFC tasks. Specifically, Hit rates (H; correctly recognising the higher concentration positioned on the left side) and False Alarm rates (F; mistakenly recognising the lower concentration positioned on the left side as the higher concentration) were calculated for *decremental* and *incremental series* separately. A decremental d′ value – d′ _(Decremental,Reference)_ – as well as an incremental d′ value – d′ _(Reference,Incremental)_ – were calculated using the equation from Macmillan and Creelman (70), for each sensory stimulus and each individual. The individual value of d′ resulted from the addition of d′_(D,R)_ and d′ _(R,I)_ for each participant and each sensory stimulus. Additionally, extreme values of d′ were corrected using the 1/(2N) rule (71). In line with the rule, extreme proportions of H and F reaching a value of 1 or 0 were replaced with 1–1/(2N) and 1/(2N), respectively, with N being the number of trials used in the experimental design. The calculations of d′_(D,R)_, d′ _(R,I),_ and d′ were performed on Excel (Microsoft office, 2018, United States).

### Statistical Analyses

Pearson’s correlations were firstly calculated to assess the relationships between continuous values of BMI and peripheral levels of ghrelin and leptin. Pearson’s correlations were also calculated between sensory measures of supra-threshold sensitivity (d′), IR and VR for each olfactory compound. Subsequently, Pearson’s and partial correlations were calculated between olfactory functions (d′, IR, and VR) and peripheral ghrelin levels, with and without BMI as a covariate. Similar analyses were then performed to assess the correlations between olfactory functions (d′, IR, and VR) and peripheral leptin levels. For all correlation analyses, the strength of the correlation observed was reported as weak, moderate, and strong for an absolute value of the correlation coefficient in the ranges of 0–0.4, 0.4–0.7, and 0.7–1, respectively (72). All statistical analyses used an α level of 0.05 for detecting significant differences, and analyses were performed on SPSS Statistics (V26 – IBM Corp., Armonk, NY, United States) and GraphPad Prism 8.0 (GraphPad Software, San Diego, CA, United States).

## Results

### Summary of Testing Measures

**FIGURE 1:**
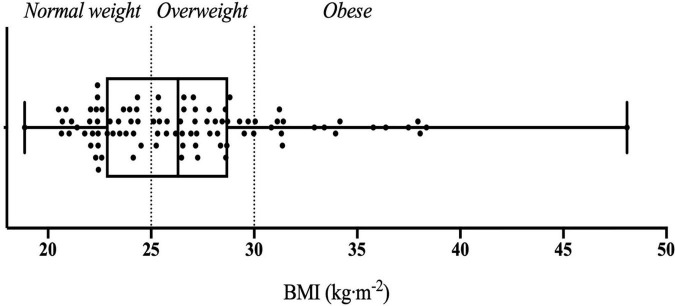
Whisker plot illustrating individuals’ BMI across three BMI groups classified as normal weight, overweight, and obese.

The present study included a total of 94 Caucasian male participants with an average age of 25.81 ± 5.74 years old, body fat percentage of 19.99 ± 5.69% and BMI of 26.78 ± 4.93 kg⋅m^–2^ (Figure 1). Table 2 summarises the mean values and standard deviations of leptin and ghrelin concentrations in periphery, as well as olfactory performances for three food-related odorants (O1, O2, and O3) across BMI groups.

**TABLE 2:** Summary of Mean ± SD values of hormonal measures and olfactory functions across BMI groups with results from One-way ANOVAs assessing the effect of BMI groups on each testing variables.

	Normal weight	Overweight	Obese	ANOVA	Total

**N**	**38**	**37**	**19**	***F*-statistics; *p-*value**	**94**
Ghrelin (pg⋅mL^–1^)	20.17 ± 18.33	13.07 ± 6.76	9.51 ± 2.62	*F*_(2,91)_ = 5.51; *p* = *0.005*	15.22 ± 13.08
Leptin (pg⋅mL^–1^)	788.76 ± 800.37	2011.63 ± 1754.01	8968.26 ± 6442.86	*F*_(2,91)_ = 46.35; *p* < *0.001*	2923.40 ± 4374.69
**Olfactory supra-threshold sensitivity**					
d′O1	2.51 ± 0.68	2.42 ± 0.97	2.40 ± 0.98	*F*_(2,91)_ = 0.15; *p* = 0.859	2.45 ± 0.86
d′O2	1.41 ± 0.67	1.49 ± 0.90	1.44 ± 0.55	*F*_(2,91)_ = 0.09; *p* = 0.912	1.45 ± 0.74
d′O3	1.42 ± 0.88	1.24 ± 0.61	1.08 ± 0.61	*F*_(2,91)_ = 1.46; *p* = 0.236	1.28 ± 0.74
**Olfactory intensity ratings**					
IRO1	26.16 ± 13.74	24.05 ± 11.39	29.39 ± 14.03	*F*_(2,91)_ = 1.08; *p* = 0.344	25.98 ± 12.93
IRO2	36.65 ± 15.67	32.08 ± 14.22	35.37 ± 18.18	*F*_(2,91)_ = 0.83; *p* = 0.441	34.60 ± 15.63
IRO3	26.15 ± 14.02	19.81 ± 11.90	22.50 ± 12.62	*F*_(2,91)_ = 2.25; *p* = 0.111	22.92 ± 13.12
**Olfactory valence ratings**					
VR O1	30.80 ± 19.57	26.57 ± 19.43	32.56 ± 16.02	*F*_(2,91)_ = 0.78; *p* = 0.459	29.49 ± 18.82
VR O2	−14.86 ± 25.35	−15.18 ± 26.50	−15.75 ± 29.33	*F*_(2,91)_ = 0.01; *p* = 0.993	−15.16 ± 26.35
VR O3	−4.24 ± 17.80	−1.91 ± 17.82	0.39 ± 15.36	*F*_(2,91)_ = 0.47; *p* = 0.623	−2.39 ± 17.25

*O1: vanilla odour, O2: potato odour, O3: dairy odour, d′: supra-threshold sensitivity (0–4), IR: intensity rating (0–100), VR: valence rating (−100 to 100).*

A total of eleven separate One-way ANOVAs examining the effect of BMI groups on each testing variables were performed. Results revealed significant main effects of BMI groups on peripheral ghrelin and leptin levels (Table 2). *Post hoc* tests, based on simple effects tests with *Bonferroni* corrections revealed that normal-weight individuals had a significantly higher level of ghrelin than overweight individuals (*p* = *0.048*) and individuals with obesity (*p* = *0.009*, see mean values in Table 2). Additionally, individuals with obesity had higher levels of leptin in periphery than normal weight (*p* < *0.001*) and overweight individuals (*p* < *0.001*, see mean values in Table 2). On the other hand, there was no significant main effect of BMI groups on olfactory measures (Table 2).

### Associations Between Peripheral Ghrelin and Leptin Levels and Body Mass Index

Pearson’s correlations were calculated to assess the relationship between continuous values of BMI and peripheral levels of ghrelin and leptin. Results revealed a significant negative correlation between peripheral ghrelin concentration and BMI (*r* = −0.302; *p* = *0.003*) and a positive and strong significant correlation between leptin and BMI (*r* = 0.755; *p* < *0.001*). Similar analyses were performed between body fat percentage measures and peripheral levels of ghrelin and leptin, which revealed comparable results (see details in Supplementary Table 1).

### Associations Between Olfactory Measures and Body Mass Index

A series of Pearson’s correlations were calculated to assess for associations between continuous values of BMI and olfactory sensory measures including supra-threshold sensitivity (d′), IR and VR for three food-related odorants. Results revealed that BMI and suprathreshold sensitivity for O3 (dairy smell) were significantly negatively correlated (*r* = −0.217; *p* = *0.036*). In contrast, sensitivities to other odorants (O1 and O2) as well as all IR and VR were not significantly correlated to BMI (*p* > 0.05, see details in in Supplementary Table 2). Similar analyses were performed between body fat percentage and olfactory measures, which revealed comparable results (see details in Supplementary Table 2).

### Links Between Olfactory Sensory Measures

**FIGURE 2:**
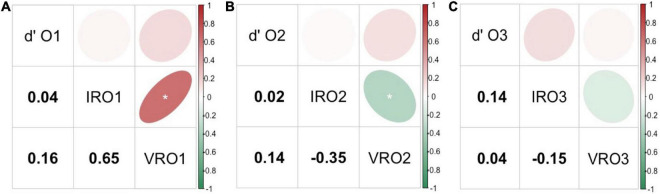
Pearson’s correlations across three types of olfactory measures: supra-threshold sensitivity (d′), intensity ratings (IR) and valence ratings (VR) for the odorants O1 **(A)**, O2 **(B)**, and O3 **(C)**. The upper part of each figure illustrates the strength of the correlation *via* an elliptical shape, and the lower part shows the correlation coefficient value for each pair of sensory measures compared. Positive correlations are represented in a red gradient and negative correlation in a green gradient. O1: vanilla odour, O2: potato odour, O3: dairy odour. Significant results are represented by *.

Pearson’s correlations were calculated between sensory measures of supra-threshold sensitivity (d′), IR and VR for each olfactory compounds tested. For all three odorants, sensitivity scores were not shown to be significantly correlated to any of the rating measures (IR and VR) (Figure 2). Regarding O1, a significantly positive and strong correlation was observed between both rating measures IRO1 and VRO1 (*r* = 0.65, *p* < *0.001*, see Figure 2A). Then, when looking at the olfactory compound O2, a significantly negative correlation was noted between IRO2 and VRO2 (*r* = −0.35, *p* < *0.001*, see Figure 2B). In contrast, IR and VR were not significantly correlated for the olfactory compound O3 (see Figure 2C).

### Relationships Between Peripheral Ghrelin Levels and Olfactory Functions

**FIGURE 3:**
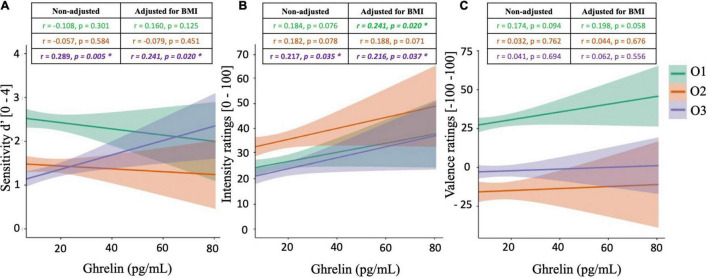
Correlation plots between peripheral ghrelin levels and olfactory supra-threshold sensitivity **(A)**, olfactory intensity ratings **(B)**, and olfactory valence ratings **(C)** of the odorants O1 (vanilla odour), O2 (potato odour), and O3 (dairy odour). All graphical plots illustrate results obtained from Pearson’s correlations (non-adjusted). Significant results are represented by *.

Pearson’s correlations were calculated between ghrelin concentrations and olfactory supra-threshold sensitivities, olfactory IR, and olfactory VR, for all three food-related odorants O1, O2, and O3 (Figure 3). These analyses revealed two significantly positive correlations, both involving the olfactory compound O3. Specifically, peripheral ghrelin levels were shown to be significantly positively correlated to d′O3 (*r* = 0.289, *p* = *0.005*, Figure 3A) and IRO3 (*r* = 0.217, *p* = *0.035*, Figure 3B). On the other hand, no correlation was observed between olfactory VR for any of the odorant tested and peripheral ghrelin levels (Figure 3C).

Subsequently, partial correlations were calculated between peripheral ghrelin levels and olfactory performances, accounting for BMI. Results from partial correlations also revealed that ghrelin concentrations were significantly and positively correlated to different measures of olfactory function, with a specific emphasis on IR. Specifically, positive correlations were observed between peripheral levels of ghrelin and IRO1 (*r* = 0.241, *p* = *0.020*) and IRO3 (*r* = 0.216, *p* = *0.037*), and a trend toward statistical significance was observed with IRO2 (*r* = 0.188, *p* = 0.071). Furthermore, a significant positive relationship was still observed between peripheral ghrelin levels and d′O3 after adjusting for BMI (*r* = 0.241, *p* = *0.020*). Finally, while ghrelin levels were not significantly correlated to olfactory VR, a tendency toward significance was noted with VRO1 (*r* = 0.198, *p* = *0.058*).

### Relationships Between Peripheral Leptin Levels and Olfactory Functions

**FIGURE 4:**
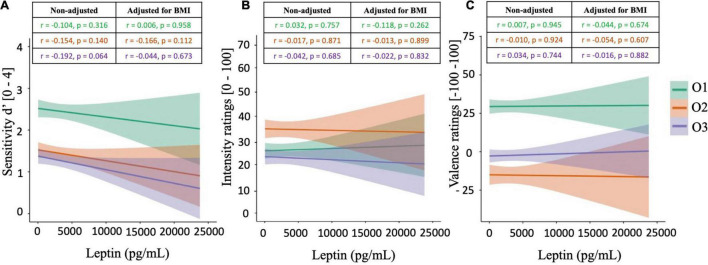
Correlation plots between peripheral leptin levels and olfactory supra-threshold sensitivity **(A)**, olfactory intensity ratings **(B)**, and olfactory valence ratings **(C)** of the odorants O1 (vanilla *odour*), O2 (potato *odour*), and O3 (dairy *odour*). All graphical plots illustrate results obtained from Pearson’s correlations (non-adjusted). Significant results are represented by *.

Pearson’s correlations were calculated between peripheral leptin levels and olfactory supra-threshold sensitivities, olfactory IR, and olfactory VR, for all three food-related odorants O1, O2, and O3 (Figure 4). Regarding results on sensitivity measures, a trend toward statistical significance was noted between d′O3 and peripheral leptin concentrations (*r* = −0.192, *p* = 0.064), but not with other odorants O1 and O2 (Figure 4A). Additionally, results did not reveal any significant correlation between peripheral leptin and olfactory IR (Figure 4B), nor VR (Figure 4C).

Subsequently, partial correlations were calculated between peripheral leptin levels and olfactory performances, accounting for BMI. Results from partial correlations revealed similar outcomes as the ones from Pearson’s correlations, with no significant correlation between peripheral leptin levels and any of the olfactory functions tested (*p* > 0.05, Figure 3). Notably, the tendency toward significance between d′O3 and peripheral leptin concentrations was no longer observed after adjusting the correlation for BMI (*r* = −0.044, *p* = 0.673).

## Discussion

The present study investigated the links between fasting individual olfactory functions and peripheral concentrations of ghrelin and leptin, two hormones highly involved in eating behaviour and previously used as biomarkers for obesity. Results from our study support the role of ghrelin in modulating individual olfactory sensitivity and intensity perception of specific food odours (i.e., *dairy* and *vanilla* odours), whereas no significant relationships were observed for leptin.

Firstly, the present study confirmed previously reported links between adiposity and hormonal balance, with individuals with obesity having lower levels of ghrelin (12, 15) and higher levels of leptin compared to normal-weight individuals (73, 74). Additionally, the present study also demonstrated that different sensory measures represent distinct phases of perception, with no evidence for correlations between olfactory sensitivity and ratings, in line with previous observations of gustation [e.g., (75)].

The present analyses showed a positive link between fasting peripheral ghrelin levels and supra-threshold sensitivities with the *dairy* odour, with additional positive relationships based on IR of the *dairy* and *vanilla* odour. The observed positive relationships to two of these odours were in line with a recent study of Uygun et al. (50), in which peripheral levels of ghrelin was correlated to odour detection scores. By contrast, no relationship was found between ghrelin and olfactory measures of the *potato* odour, regardless of controlling for BMI. The inconsistent results across olfactory compounds imply an odour-specific relationship of ghrelin effects. Similarly, Trellakis et al. (49) investigated the link between ghrelin levels and olfactory VR of six odours, and observed only one significant correlation (with black pepper oil). Moreover, studies using a neutral odour of *n-butanol* failed to observe a ghrelin-olfaction link (47–49), while other studies using food-related odours did (39, 48). These previously observed inconsistencies may be attributed to choices of neutral versus food-related odours. The current findings further highlight discrepant results across different food odours (e.g., *vanilla* versus *potato* odours).

It is important to note that the observed ghrelin-olfaction links were subject to the sensory measure. Specifically, significant results were found with supra-threshold sensitivity and intensity rating (to specific odours). However, none of the analyses based on odour valence showed significance (although correlation for the vanilla odour was close to significance after BMI being controlled). Ghrelin links to odour valance had been tested in two separate studies, with findings remaining controversial (48, 49). For instance, Trellakis et al. (49) found that ghrelin was significantly correlated with valence to an odour of black pepper oil, but did not find evidence for relationships based on odour discrimination nor identification. Recent research has found that different types of sensory functions, such as intensity and valence perception, involve distinct brain regions (29). Specifically, evidence has shown that odour intensity perception activates the amygdala, while valence emerges from activations of the orbitofrontal cortex (76). Moreover, Russo et al. (42) demonstrated that ghrelin injections were able to modulate rat’s sense of smell, by altering olfactory transduction from the mitral cells to the amygdala – a cortical area thought to be important for intensity perception. In this context, our study offers new behavioural data on close links between ghrelin and odour intensity perception in humans. Additionally, these findings point to the importance of considering specific sensory functions when evaluating links to ghrelin levels.

In general, the present study failed to detect strong associations between leptin levels and olfactory measures, which was in line with findings from Uygun et al. (50). In contrast, numerous previous investigations have reported either positive (49, 58) or negative associations between leptin levels and olfactory sensitivities (51, 54, 55, 58, 59). In addition, one study reported negative correlations between leptin and odour valence (49). Previously, obesity has been linked to increased leptin levels and declined olfactory functions [cf. (7, 77)]. Building upon these findings, we originally proposed that leptin should be negatively correlated to odour sensitivity. Notably in our results, correlation between leptin and sensitivity to the *dairy* odour was close to significance (*p* = 0.06) but increased drastically with correlations controlling for BMIs (*p* = 0.67). Such change in correlation coefficients suggests that the link between leptin and odour sensitivity was prominently mediated by individual BMIs. In this tripartite relationship, BMI plays a more substantial role in linking leptin and olfactory sensitivities. While future studies are needed to confirm this proposal, our data appear to suggests that leptin and olfactory performance are not directly linked.

The current findings point to the important role of olfactory processing in mediating ghrelin influences on food intake (78). Elevated peripheral ghrelin levels have been previously observed in individuals experiencing food reward anticipation, which is characterised by an increased cerebral activity in reward-related areas, such as the orbitofrontal cortex (79, 80). Furthermore, evidence suggests that peripheral ghrelin concentration following consumption of energy-dense food maintains at a high level in sated healthy-weight individuals, in contrary to the expected post prandial ghrelin profile (5, 22). Our study has found that individuals with fasting elevated peripheral ghrelin levels show heightened olfactory supra-threshold sensitivity and intensity perception for specific food odours, which may facilitate food-seeking behaviour. This finding may be related to neural evidence for convergence of ghrelin and sensory functions, as evidence indicates that sensory food cues and systemic ghrelin administration activate the same subset of hypothalamic neurons (81). These connexions directly imply a co-action of ghrelin signalling and olfactory perception, resulting in increased appetite and food intake. Notably, recent research indicated that plasma ghrelin only targets specific brain regions, including the hypothalamus, the mesolimbic pathway, as well as the OB [cf. (82)]. The latter brain area, which is directly involved in olfactory coding processes (25, 27), was shown to be one of the regions with the highest uptake of systemically injected ghrelin (83). Inversely, a recent study from Riera et al. (84) suggested that olfactory activity directly modulates fat mass and hormonal alteration associated with obesity. Therefore, findings from both previous research and the present study support the concept of bidirectional relationship between olfactory perception and metabolic regulation through ghrelin signalling. Overall, these findings bring new insights into understanding individual susceptibility to overeating and obesity.

A limitation of the present study was that no serine protease inhibitor was added to the K_2_ EDTA blood samples before measuring acyl ghrelin concentrations. The addition of serine protease inhibitor is typically used to limit the degradation of acyl ghrelin due to deacylation (85, 86). A common serine protease inhibitor is aprotinin, which has been widely used as a standardized procedure to prevent from acyl ghrelin degradation in previous research (87). The absence of a protease inhibitor in the present experiment may explain the rather low concentrations of acyl ghrelin reported in this study. However, Blatnik and Soderstrom (86) observed no significant difference in the degree of acyl ghrelin degradation, when plasma samples were stored in K_2_ EDTA tubes only or in K_2_ EDTA tubes containing aprotinin, with both storage leading to an approximate loss of 50% of acyl ghrelin. Therefore, concentrations of acyl ghrelin presented in this study should be interpreted with a certain degree of caution.

## Conclusion

Overall, the present study investigated links between individual olfactory functions and two major peptide hormones. The findings point to the important role of ghrelin in influencing olfactory performances. Specifically, strong relationships were observed between peripheral ghrelin levels and olfactory functions, in particular intensity perception, of specific food odours. Furthermore, peripheral leptin levels were not linked to any of the tested olfactory performances. Results from the present study brings new data supporting the role of olfaction in mediating hormonal effects on eating.

## Data Availability Statement

The original contributions presented in the study are included in the article/Supplementary Material, further inquiries can be directed to the corresponding author.

## Ethics Statement

The studies involving human participants were reviewed and approved by the University of Otago Human Ethics Committee (Health). The patients/participants provided their written informed consent to participate in this study.

## Author Contributions

RG, SA, and MP contributed to conception and design of the study. RG and SA organized the database. RG performed the statistical analysis and wrote the first draft of the manuscript. MP and IO edited the manuscript. RG, SA, IO, and MP contributed to manuscript revision, read, and approved the submitted version.

## Conflict of Interest

The authors declare that the research was conducted in the absence of any commercial or financial relationships that could be construed as a potential conflict of interest.

## Publisher’s Note

All claims expressed in this article are solely those of the authors and do not necessarily represent those of their affiliated organizations, or those of the publisher, the editors and the reviewers. Any product that may be evaluated in this article, or claim that may be made by its manufacturer, is not guaranteed or endorsed by the publisher.
