# Supplementary material for: Testing Links of Food-Related Olfactory Perception to Peripheral Ghrelin and Leptin Concentrations
Source: Front Nutr. 2022 May 11;9:888608. doi: 10.3389/fnut.2022.888608 (PMC9130723; doi:10.3389/fnut.2022.888608)
Supplement: Supplementary file 1 [file Table_1.DOCX]

Supplementary tables

Table 1 Correlation coefficients r and p-values of Pearson’s correlations calculated between body fat percentages (BFP) and peripheral levels of ghrelin and leptin. Significance is indicated by italic values (p ≤ 0.05).

|  |  | Ghrelin | Leptin |
| --- | --- | --- | --- |
| BFP (%) | r | *-0.35* | *0.70* |
|  | p-value | *< 0.001* | *< 0.001* |

Table 2 Correlation coefficients r and p-values of Pearson’s correlations calculated between body adiposity variables (BMI and BFP) and olfactory functions (suprathreshold sensitivity, intensity ratings, and valence ratings) for three odorants (O1,O2,and O3). Significance is indicated by italic values (p ≤ 0.05).

|  | | Suprathreshold sensitivity | | | Intensity ratings | | | Valence ratings | | |
| --- | --- | --- | --- | --- | --- | --- | --- | --- | --- | --- |
|  | | d´ O1 | d´ O2 | d´ O3 | IR O1 | IR O2 | IR O3 | VR O1 | VR O2 | VR O3 |
| BMI | r | -0.143 | -0.060 | *-0.217* | 0.144 | -0.011 | -0.037 | 0.048 | 0.034 | 0.059 |
|  | P-value | 0.169 | 0.568 | *0.036* | 0.167 | 0.917 | 0.725 | 0.648 | 0.747 | 0.575 |
| BFP | r | -0.132 | -0.096 | *-0.263* | 0.142 | -0.059 | -0.124 | -0.032 | -0.067 | 0.134 |
|  | P-value | 0.206 | 0.359 | *0.011* | 0.173 | 0.571 | 0.234 | 0.758 | 0.520 | 0.196 |
| Note: O1: vanilla smell, O2: potato smell, O3: dairy smell; BMI: body mass index, BFP: body fat percentage | | | | | | | | | | |
